# Litchi seed extracts diminish prostate cancer progression via induction of apoptosis and attenuation of EMT through Akt/GSK-3β signaling

**DOI:** 10.1038/srep41656

**Published:** 2017-01-30

**Authors:** Hongwei Guo, Hua Luo, Hebao Yuan, Yudui Xia, Pan Shu, Xin Huang, Yi Lu, Xia Liu, Evan T. Keller, Duxin Sun, Jiagang Deng, Jian Zhang

**Affiliations:** 1 Center for Translational Medicine, Guangxi Medical University, 22 Shuangyong Road, Nanning 530021, China; 2 Key Laboratory of Longevity and Aging-related Disease, Chinese Ministry of Education, 22 Shuangyong Road, Nanning 530021, China; 3 College of Pharmacy, Guangxi University of Chinese Medicine, 179 Mingxiu Dong Road, Nanning 530001, China; 4 Department of Pharmaceutical Sciences, College of Pharmacy, University of Michigan, 1600 Huron Parkway, Ann Arbor, MI 48109, USA; 5 Xinxiang Central Hospital of Henan, 56 Jinsui Road, Xinxiang 453000, China; 6 Department of Urology and Pathology, School of Medicine, University of Michigan, 2800 Plymouth Road, Ann Arbor, MI 48109, USA; 7 Southern University of Science and Technology, School of Medicine, 1088 Xueyuan Blvd., Nanshan District, Shenzhen, 518055, China

## Abstract

Litchi (*Litchi chinensisSonnnerat, Sapindaceae*), known as Chinese Cherry, is a subtropical fruit tree originating from southern China. Litchi seed extracts have diverse pharmacological effects, including anticancer. However, its anticancer effects and mechanisms on prostate cancer have not been determined. In this study, we used n-butyl alcohol extract of Litchi seed (NLS) to treat prostate cancer PC3, DU145, RM1 and C4-2B cells. NLS induced a significant decrease in cell viability and clonogenic growth in a dose-dependent manner. NLS induced cell apoptosis and cell cycle G1/S phase arrest by inactivating Akt signaling pathway, which were associated with activation of mitochondrial caspase-dependent apoptotic cascades, up-regulation of cyclin-dependent kinase (CDK) inhibitors p21 and p27, and inhibition of correlated cyclin/CDK network. In addition, NLS treatment significantly decreased cell migration and invasion via phenotypic inversion of EMT, correlated with increased expression of E-cadherin and β-catenin, and decreased expression of vimentin and snail, which is partially attributed to inhibiting Akt/GSK-3β signaling pathway. Finally, PC3 xenograft nude mice treated with NLS *in vivo* showed a significant decrease in tumor size without toxicity. These findings suggest that NLS has potential for development into a safe and potent alternative therapy for prostate cancer patients.

Prostate cancer (PCa) is the second most frequently diagnosed cancer in men worldwide, with 1.1 million new cases occurred in 2012^1^. In developed countries, however, PCa is the most frequently diagnosed cancer and the second leading cause of cancer-related death among men^1^,^2^,^3^. There will be 1.7 million new cases and 499, 000 new deaths by 2030 due to the growth and aging of the global population^4^. The search for alternative and affordable treatments is thus of great significance and medicinal plants may present a great opportunity for future research on new anticancer agents.

Litchi (*Litchi chinensisSonnnerat, Sapindaceae*), known as Chinese Cherry, Leechee, Lichee, Litchi, Lychee, Mountain Lychee, and Water Lychee, is a subtropical fruit tree originating from southern China and is cultivated in semitropical areas worldwide for its palatable sweet fruits^5^,^6^. Currently, Litchi pulp is mainly consumed freshly, but Litchi seed is discarded and underutilized. In China, Litchi seed is used to remove pathogenic cold and release stagnant humor. The seeds also serve as an analgesic agent relieving neuralgia, orchitis, testicular swelling, hernia, gastralgia, lumbago, abdominal pain and so on^7^,^8^. The decoctions of Chinese herbal formula containing Litchi seeds are used as indigenous remedy for urologic neoplasms including prostate cancer, bladder cancer and renal carcinoma^9^,^10^. In the past few decades, many compounds have been isolated from Litchi seeds, including flavonoids, proanthocyanidins, volatile constituents, sterols, fatty acids, amino acids, phenolic acids, dihydrochalcones, coumarins, sesquiterpenes, and triterpenes etc^6^. Pharmacological studies have shown that Litchi seeds have multiple bioactivities such as anticancer, anti-oxidant, anti-inflammation, anti-hyperlipidemia, antivirus, anti-diabetes, antibacterial, hypoglycemic and immuno-regulation^6^,^8^,^11^,^12^. Recently, several studies have revealed that Litchi seed extracts exhibit anti-cancer activity towards lung, cervical, colorectal, liver and nasopharyngeal cancers^7^,^8^,^11^,^13^,^14^. However, there is no report to demonstrate the effects and mechanisms of Litchi seed extracts on PCa. Here, we investigated the effects of n-butyl alcohol extract of Litchi seed (NLS) on PCa PC3, DU145, RM1 and C4-2B cell lines and explored its potential mechanisms.

## Results

### Phytochemical analysis of NLS

**Figure 1:**
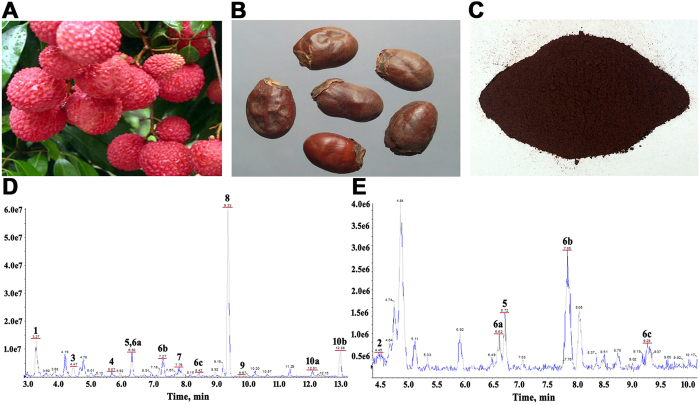
Phytochemical fingerprint of NLS. (**A**) Litchi fruit. (**B**) Litchi seeds. (**C**) n-butyl alcohol extract of litchi seeds (NLS). (**D**) The base peak chromatogram of NLS by HPLC-MS in negative mode. (**E**) The base peak chromatogram of NLS by HPLC-MS in positive mode.

We analyzed the phytochemical ingredients of NLS using HPLC-MS. Figure 1 showed the base peak chromatogram of NLS by HPLC-MS. All compounds were characterized by the interpretation of their mass spectra obtained by the MS/MS and were further aligned with published data from literatures. The 13 compounds presented were summarized in Table 1, including flavonoids, phenols, proanthocyanidins, sesquiterpenes, fatty acids, and so on.

### NLS inhibited growth and viability of PCa cells

**Figure 2:**
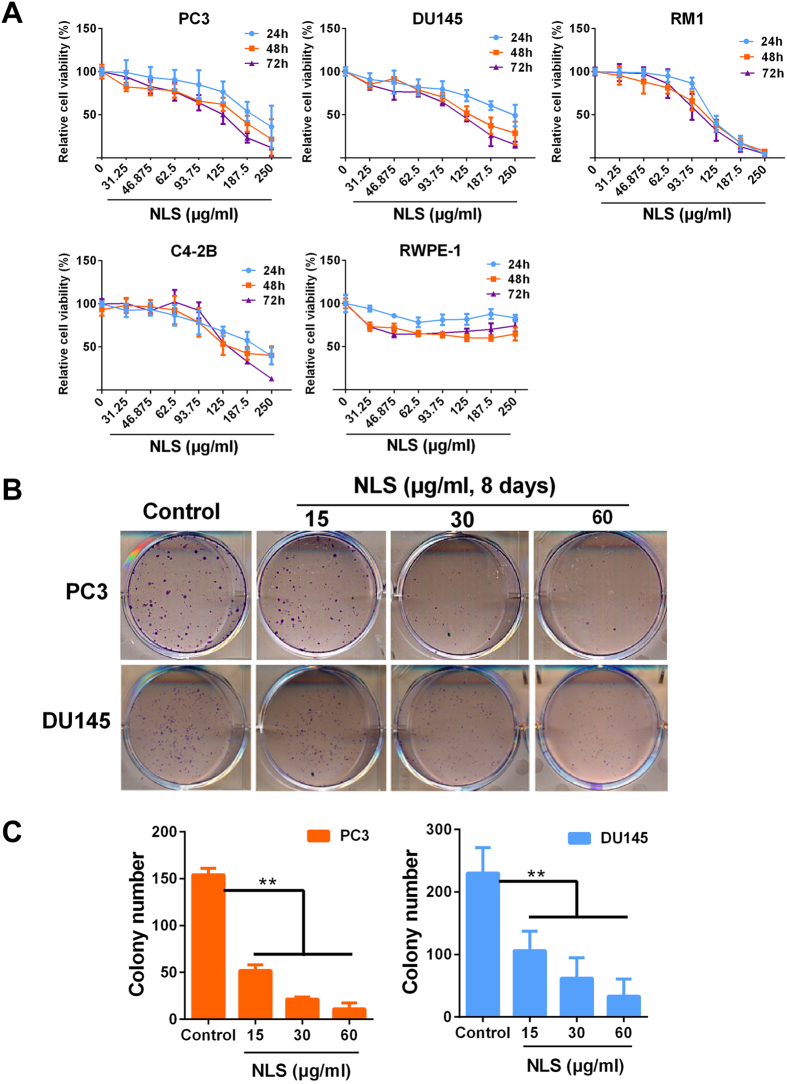
Effect of NLS on the viability of prostate cancer cells. (**A**) Prostate cancer cell lines PC3, DU145, C4-2B, RM1, and immortalized human normal prostatic epithelial cell line RWPE-1 were treated with NLS at concentrations of 0 to 250 μg/ml in triplicates for 24, 48 and 72 h. Cell viability was measured by MTS assay. (**B**) Evaluation of the clonogenic growth potential of PC3 and DU145 cells treated with NLS for 8 days. At the end of incubation, colony formation was observed by staining with crystal violet. (**C**) Bar plot of the average colony number of PC3 and DU145 cells. Similar results were obtained in 3 independent experiments. Data are expressed as mean ± SD. Compared with control group: **p < 0.01.

To investigate the effect of Litchi seed extract on proliferation of PCa cells, PC3, DU145, C4-2B, and RM1 cells were treated with four fractions of Litchi seed extract (0 to 250 μg/ml) for 24, 48 and 72 h, and viability was determined by MTS assay, normal prostatic epithelial cell line RWPE-1 was used as controls. We observed that the n-butyl alcohol and ethyl acetate fractions inhibited cell growth in a dose-dependent manner. More importantly, n-butyl alcohol fraction (NLS) was more potent in inhibiting proliferation of PCa cells compared to the ethyl acetate fraction (Fig. 2A and Supplementary Fig. S1). The IC_50_ values of NLS for PC3 cell were 147.3, 128.14 and 111.97 μg/ml; for DU145 cell, 114.31, 104.09 and 105.23 μg/ml; for RM1 cell, 116.52, 107.10 and 101.41 μg/ml; and for C4-2B cell, 119.97, 105.46 and 122.78 μg/ml for 24 h, 48 h and 72 h respectively. However, the viability of normal prostatic epithelial cell line RWPE-1 was not inhibited by NLS and IC_50_ values were over 250 μg/ml (Fig. 2A).

In addition, clonogenic assay was performed to further validate the inhibiting effect of NLS on PCa cells. As shown in Fig. 2B,C, PC3 and DU145 cells treated for 8 days with NLS at 15, 30 and 60 μg/ml showed a significant dose-dependent inhibition of colony formation compared to dimethylsulfoxide (DMSO)-treated controls. These results demonstrated that NLS showed potent growth inhibition towards PCa cells.

### NLS Induced the Apoptosis of Prostate Cancer Cells

**Figure 3:**
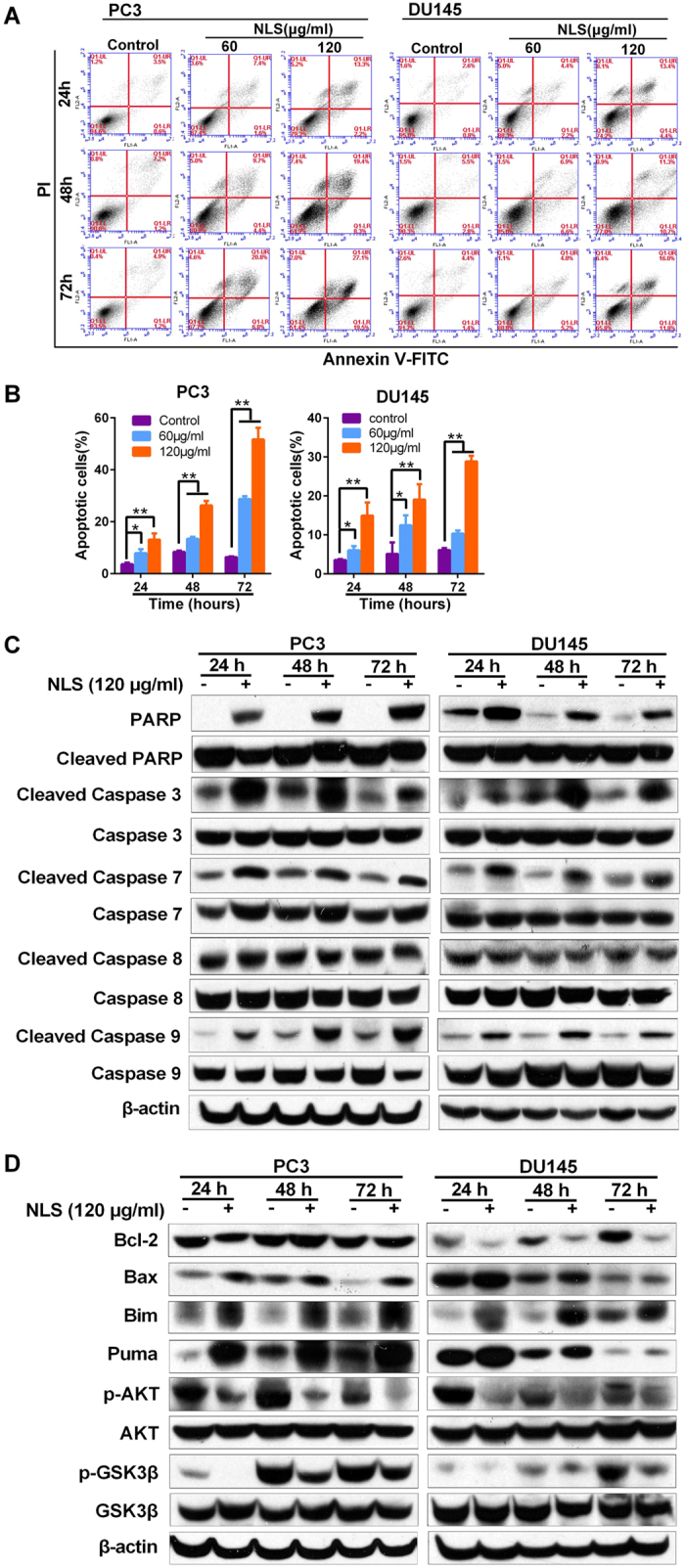
NLS induced apoptosis of PC3 and DU145 cells. (**A**) PC3 and DU145 cells were treated with NLS (60 and 120 μg/ml) for 24, 48 and 72 h and the cell apoptosis was evaluated by flow cytometry after propidium iodide (PI) and Annexin V staining. The cell populations were discriminated in each quadrant as viable cells in the lower left (annexin V negative/PI negative), early apoptotic cells in the lower right (annexin V positive/PI negative), late apoptotic cells in the upper right (annexin V positive/PI positive), and necrotic cells in the upper left quadrant (annexin V negative/PI positive). (**B**) The quantitative data of panel A. The percentages of Annexin V-positive cells are shown. *p < 0.05; **p < 0.01. (**C**) Apoptosis-related markers such as poly ADP ribose polymerase (PARP) cleavage and caspases activities were detected by western blotting in PC3 and DU145 cells. (**D**) Bcl-2 family of proteins and Akt and GSK-3β activities were detected by western blotting in PC3 and DU145 cells.

To determine the primary driver for the decreased cell viability, we analyzed the apoptosis in NLS treated prostate cancer cells with flow cytometry. We observed significant dose- and time-dependent increase in the population of apoptotic cells in both PC3 and DU145 cell lines when treated with NLS (Fig. 3A,B). The apoptotic cell populations in PC3 and DU145 cells treated with high-dose NLS for 72 h, were increased to 55.3% and 28.2%, respectively, compared to that in the DMSO-treated control cells (6.4% and 5.8%).

To further explore the mechanisms underlying NLS-induced apoptosis of prostate cancer cells, we detected the expression of a panel of proteins involved in cellular apoptosis using western blotting. NLS treated cells showed increased expression of activated Caspase-3, associated with PARP cleavage (Fig. 3C), further validating that apoptosis was the primary mechanism of NLS-induced growth inhibition in PCa cells. To investigate whether NLS induced apoptosis is mediated through activation of extrinsic or intrinsic pathways, we next examined the expression of Caspases-8 and Caspases-9 in NLS treated cells. We found NLS led to an increase of cleaved Caspase-9 in cells associated with enhanced expression of cleaved Caspase-7, yet failed to induce the changes of caspase-8, which is involved in the extrinsic apoptotic pathway (Fig. 3C). These results suggested that NLS activated the intrinsic apoptotic pathway.

Next, we assessed the effect of NLS on the Bcl-2 family of proteins involved in apoptosis. Both PC3 and DU145 cells treated with NLS showed an increase in the expression of pro-apoptotic Bax, Bim and Puma. Moreover, NLS decreased the expression of anti-apoptotic Bcl-2 protein in DU145 cells. Furthermore, NLS inhibited significantly the phosphorylation of Akt and GSK-3β in both PC3 and DU145 cell lines (Fig. 3D), which are consistent with the roles for Akt and GSK-3β in the regulation of apoptosis. These data demonstrated that NLS induced cell apoptosis in prostate cancer cells by inactivating Akt signaling pathway and activating mitochondrial caspase-dependent intrinsic apoptotic cascades.

### NLS Induced Cell Cycle Arrest in Prostate Cancer Cells

**Figure 4:**
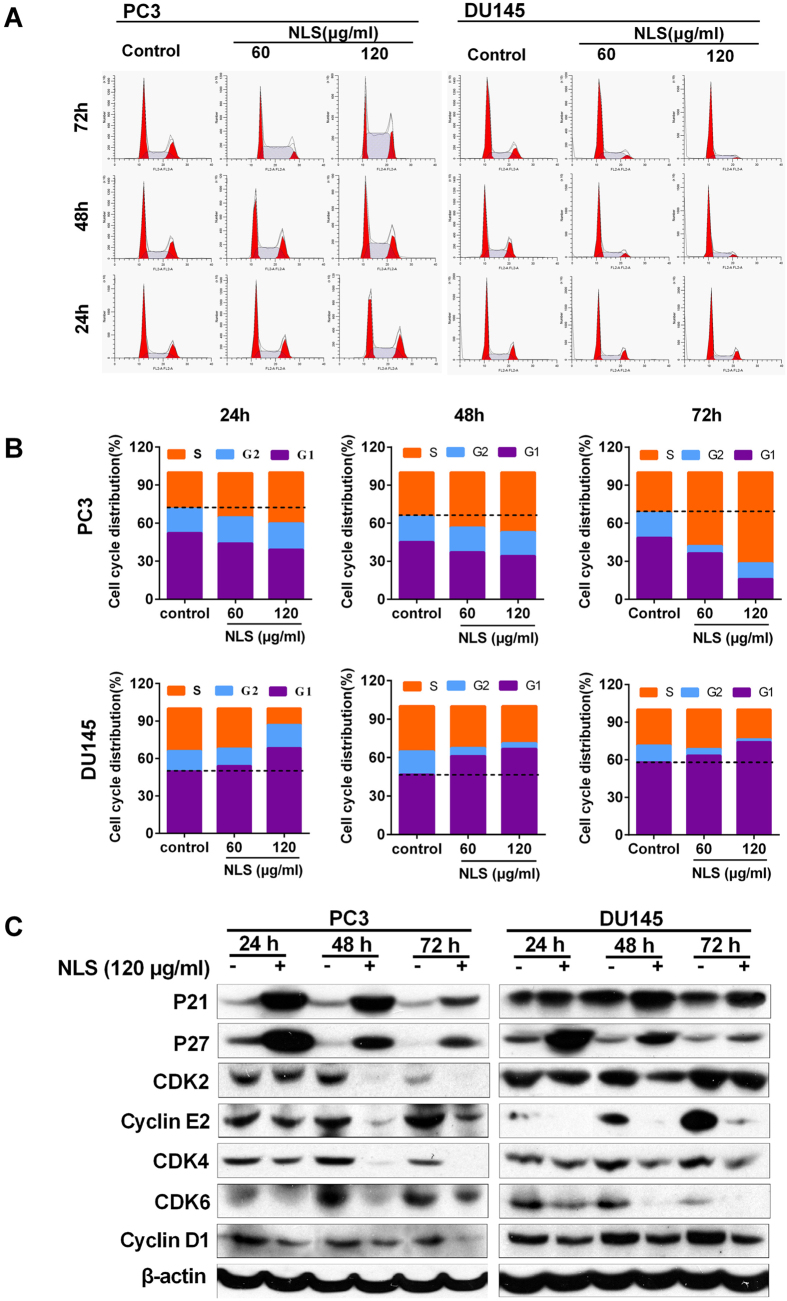
NLS arrested cell cycle progression at the G1/S phase. (**A**) PC3 and DU145 cells were treated with 60 and 120 μg/ml NLS for 24, 48, and 72 h, and the cell cycle distribution was evaluated after propidium iodide (PI) staining. Data are representative of three independent experiments. (**B**) Bar plot of the cell cycle distribution of PC3 and DU145 cells. (**C**) Expression of cell-cycle-related proteins in PC3 and DU145 cells was determined by western blotting.

Interestingly, we observed that NLS induced cell cycle arrest in prostate cancer cells as well. Propidium iodide (PI) staining for PC3 cell cycle progression showed that the majority of the cells were arrested in the S phase (Fig. 4A,B) after being treated with NLS. The increase of S phase cell population in PC3 was accompanied by a simultaneous decrease in the G1 phase cell population. In the DU145 cells, NLS treatment led to a significant increase in the proportion of cells in the G1 phase accompanied by a simultaneous decrease in the G2 phase cell population (Fig. 4A,B). Therefore, the inhibitory effects of NLS on cell proliferation and clonogenicity might partially attribute to cell cycle arrest at G1/S phase.

To determine which protein molecules might be responsible for NLS-induced G1/S arrest, we examined the levels of several cell cycle regulators during the G1/S transition. Western blotting results showed that NLS treatment elevated levels of the CDK inhibitors p21 and p27, decreased levels of CDK2, 4, 6 and cyclinD1, E2 in PC3 and DU145 cells compared with those in control cells (Fig. 4C). Taken together, our data demonstrated that NLS induced cell cycle arrest at G1/S phase through downregulation of cyclin-dependent kinases (Cdks) and upregulation of CDK inhibitors.

### NLS Reduced migration and invasion capabilities in PC3 and DU145

**Figure 5:**
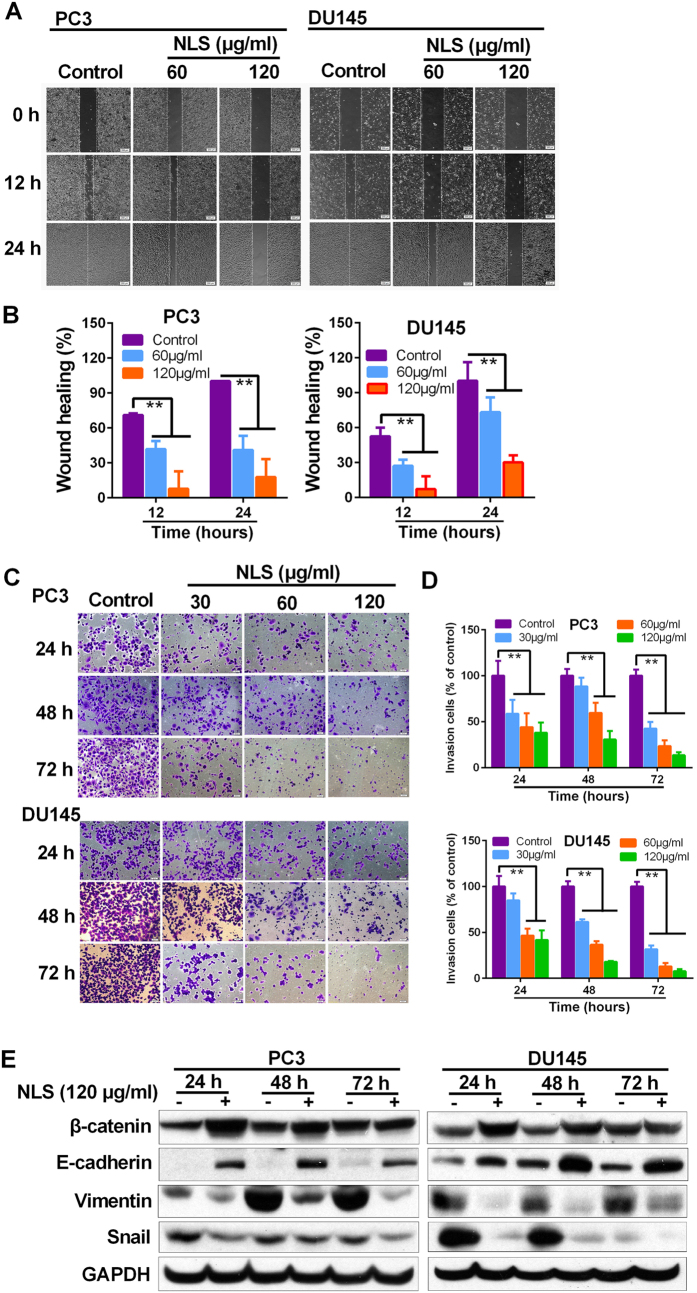
Effects of NLS on the migration and invasion capabilities in PC3 and DU145 cells. (**A**) Wound-healing assay was used to examine cellular migration. PC3 and DU145 cells were allowed to grow into full confluence in 6-well plates, and then a wound was created with a pipette tip. NLS was added to the well and images were obtained using a microscope at 0, 12 and 24 h. Three independent experiments were examined and representative images were presented. (**B**) Quantification of the average wound healing degree of PC3 and DU145 cells. (**C**) Invasiveness of PC3 and DU145 cells that underwent NLS treatment was determined in transwell invasion assay. PC3 and DU145 cells, after 24, 48 and 72 h pretreatment with NLS (30, 60 and 120 μg/ml), were added in the top chamber and allowed to invade for 22 h. Crystal violet-stained cells represent the fraction of cells that migrated from the top to the bottom chamber of the membrane. Three independent experiments were examined and representative images were presented. (**D**) Quantification of invaded PC3 and DU145 cells in the bottom chamber. Data are expressed as mean ± SD. Compared with control group: *p < 0.05, **p < 0.01. (**E**) Expression of EMT-related proteins in PC3 and DU145 cells was determined by western blotting.

Metastasis remains the leading cause for mortality in all cancer patients, including prostate cancer. To determine whether NLS may inhibit metastasis in PCa cells, we performed cell migration and invasion assays using PC3 and DU145 cells. As shown in Fig. 5, NLS, in a time- and dose-dependent manner, significantly inhibited tumor cell migration (Fig. 5A,B), and invasion (Fig. 5C,D) in both PC3 and DU145 cells. In the control group, adjacent cells migrated toward the scratched space on plates and the gap was completely closed up as time passed, whereas cells treated with NLS were not able to move toward the wounded area and left an obvious gap (Fig. 5A). We also noted that the NLS remarkably reduced the number of invaded cells stained with crystal violet compared to the control groups (Fig. 5C,D). These results indicated that NLS had metastatic inhibitory effects on human PCa cells.

To explore the mechanism of NLS on anti-metastasis, we detected the expression of β-catenin, E-cadherin, Vimentin and Snail in PC3 and DU145 cells, which are known factors involved in epithelial-mesenchymal transition (EMT) and cancer metastasis. Western blotting results showed that the expression of E-cadherin was significantly up-regulated by NLS, and the level of β-catenin was also increased after 24 h and 48 h of NLS treatment. On the contrary, the expression of Vimentin and Snail was remarkably down-regulated in the NLS-treated cells compared to control cells (Fig. 5E). These results indicated that NLS reduced migration and invasion capabilities in PCa cells via phenotypic inversion of EMT.

### NLS inhibited growth of PC3 xenografts in nude mice

**Figure 6:**
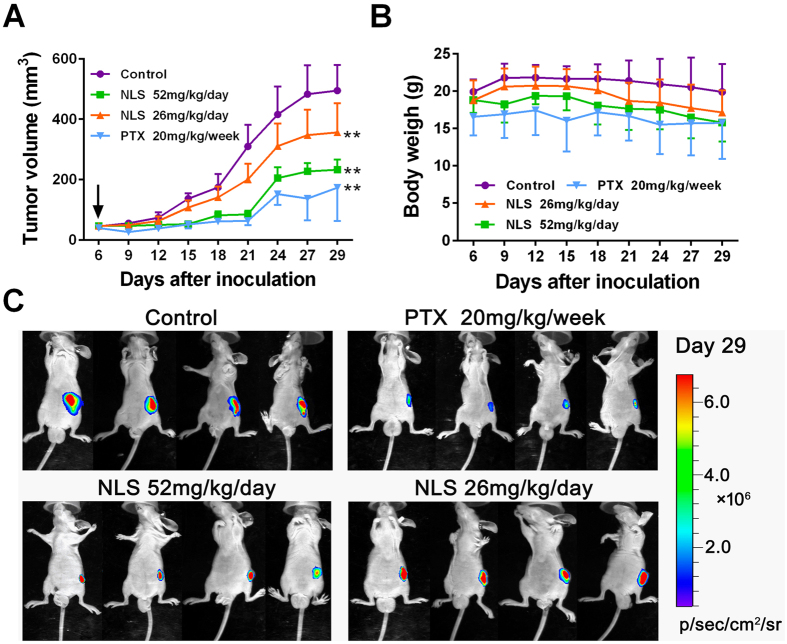
NLS inhibited tumor growth in human PC3 xenograft nude mouse model. Nude mice (*n* = 10) implanted with PC3^-luc^ cells (2 × 10^6^/mouse) by subcutaneous injection were treated for 24 consecutive days orally with NLS (52 and 26 mg/kg/day). Paclitaxel (PTX, 20 mg/kg), used as reference drug for positive control, was administered intraperitoneally once per week. Treatment was started 6 days after cell injection with the average tumor volume of about 50 mm^3^ for the treatment and control groups. (**A**) Tumor growth curves in treatment and control groups. Data are expressed as mean ± SD. Compared to control group: **p < 0.01. (**B**) Body weight measurements of mice in different groups throughout the treatment course. There is no significant loss of body weight in NLS treatment groups compared to that of control group. (**C**) Bioluminescence imaging of PC3 xenograft tumors in different groups at the end of experiments.

To evaluate the anti-PCa activity of NLS *in vivo*, we established human PC3 xenograft in nude mice by subcutaneous injection of PC3^-luc^ cells. The tumor volume and body weight were examined to evaluate the effects of the treatment. After 24 consecutive days treatment with NLS, xenografts of both NLS-treated groups had smaller tumor sizes compared to those in control group (Fig. 6A,C). As shown in Fig. 6A, NLS shrunk tumor growth to approximately 52.99% at the dose of 52 mg/kg and to 28.01% at the dose of 26 mg/kg compared to the vehicle control on Day 29. In addition, there was no significant loss of body weight during the NLS treatment (Fig. 6B), suggesting that NLS was not overtly toxic. The bioluminescence imaging of PC3 xenograft tumors in different groups at the end of experiments showed consistent results (Fig. 6C). Our results demonstrated that NLS efficiently inhibited tumor growth in PC3 xenograft models without major toxicity.

## Discussion

PCa is the most commonly diagnosed solid tumor and the second leading cause of cancer related death in men in western countries^3^. Despite remarkable advances in early diagnosis, it often escapes timely detection. Mortality remains high in advanced PCa due to relapse after androgen deprivation therapy and chemotherapy. Thus, effective treatment strategies are urgently needed. Alternative therapies using oriental herbal formulae, such as PC-SPES, KMKKT and TBS-101 as well as herbal extract components suggest new approaches to the management of anti-PCa^15^,^16^. Since carcinogenesis is a multistage process, it could be problematic by using a single agent. Therefore, the synergistic interactions among the active ingredients present in plant-based extracts may help to protect against cancer.

Litchi seeds have been used alternatively in traditional Chinese medicine for the treatment of urologic neoplasms such as prostate cancer, bladder cancer and renal carcinoma etc^9^,^10^. Previous studies have indicated Litchi seed extract possesses anti-cancer activities against various tumor cells, including lung, cervical, colorectal and liver cancer^6^, yet its anti-PCa potential has not been assessed. We employed HPLC-MS to detect the ingredients of NLS and found the presence of flavonoids, phenols, proanthocyanidins, sesquiterpenes and fatty acids in NLS (Table 1), which are consistent with published reports^17^,^18^,^19^,^20^. Xu *et al*. concluded that Litchi seeds are mainly composed of proanthocyanidins and lichitanin A^20^. One major structure of lichitanin is epicatechin that exhibited strong anti-cancer effect *in vitro*^21^, suggesting the potential role of NLS on cancer prevention and treatment. It is also well known that plant-based extracts rich in proanthocyanidins have been regarded as nutrients with cancer preventive characteristics^22^. Both flavonoids and phenols usually have a wide range of pharmacological activities, such as anti-cancer, anti-inflammatory, due to their antioxidant property^23^,^24^. These findings emphasized the therapeutic potential of NLS, likely attributable to the presence of proanthocyanidins, flavonoids and phenols.

In the present study, we demonstrated that NLS inhibited the growth of four PCa cell lines (PC3, DU145, RM1 and C4-2B) in a dose-dependent manner using MTS assays (Fig. 2A). Moreover, clonogenic assay further validated the inhibiting effect of NLS on PCa cells (Fig. 2B,C). Meanwhile, NLS exhibited the minimal toxicity on the viability of normal prostatic epithelial cell RWPE-1 (Fig. 2A), suggesting selective activity of NLS against malignant cells. Moreover, we recapitulated NLS’s anti-tumor efficacy *in vivo*, and there was no significant decrease in body weight and other associated adverse effects in mice during its treatment (Fig. 6). These findings imply NLS could be developed into a safe and potent alternative therapy for PCa patients.

We then systematically evaluated the molecular mechanisms of the anti-cancer activity of the NLS against prostate cancer cells. Previous studies showed that Litchi seeds extract could induce significant apoptosis or cell cycle arrest in hepatoma cells^13^, colorectal carcinoma cells^7^ and sarcoma cells^25^. Our data confirmed that NLS inhibited PC3 and DU145 via both inducing apoptotic cell death and arresting cell in G1/S phases, as demonstrated by flow cytometry analysis (Figs 3 and 4).

It is well known apoptosis may be executed via the extrinsic pathway or through an intracellular cascade of events that involves mitochondria. In this case, caspases, a family of cysteine proteases, are the central regulators of apoptosis. Activation of initiator caspases, such as caspase-8/9 cleave and activate downstream effector caspases (including caspase-3/7), which in turn execute apoptosis^26^. The members of the Bcl-2 family also play an essential role in regulation of apoptosis^27^. Some family members promote apoptosis (e.g., Bax and Bim), while others inhibit it (e.g., Bcl-2)^28^,^29^. In the present study, we found that treatment of NLS increases the activities of caspase-3/7/9, and PARP, enhances the expression levels of Bax, Bim and Puma, down-regulates the expression level of Bcl-2, yet fails to induce the changes of caspase-8 (Fig. 3C,D), which characterizes the extrinsic pathway of apoptosis. These results suggest that NLS induces cell death in PCa cells through the mitochondrial apoptotic pathway.

To determine which protein might be responsible for NLS-induced G1/S arrest, we first examined the levels of several cell cycle regulators during the G1/S transition. In the late G1 phase, cyclin D-CDK4/6 kinase complex initiates phosphorylation of Rb and its release from E2F results in the progression of G1/S transition. This process is enhanced by cyclin E-CDK2 complex, but inhibited by cyclin-dependent kinase inhibitors p21and p27^30^,^31^. Strikingly, we observed that expression of cyclinD1, cyclin E2 and CDK 2/4/6 was decreased while expression of p21 and p27 was increased in NLS treated cells compared to control cells (Fig. 4C). Our results suggest that NLS induces G1/S arrest through increasing the levels of p21 and p27 and decreasing the levels of cyclin D1, cyclin E2, and CDK2/4/6.

EMT is a crucial step in tumor progression and plays vital roles in cancer invasion and metastasis. In general, increased migration and invasion are positively correlated with EMT, which is characterized by repression of epithelial markers (e.g., E-cadherin) and induction of mesenchymal markers (e.g., Vimentin and Snail)^32^. β-catenin, one of the components of the adherens junction, binds directly to E-cadherin to act in cell-cell adhesion. Loss of functional E-cadherin, and thus of adherens junction-mediated cell-cell contacts was reported to be the first step of tumor invasion and metastasis in various cancers^33^. Snail encoded by SNAI1/SNA gene binds to the promoter of E-cadherin gene and represses its transcription^34^. Knockdown of Snail inhibited tumor cell invasion and migration^35^. In this study, we observed that NLS obviously increases levels of E-cadherin and β-catenin, and decreases levels of Vimentin and Snail in both PCa cells (Fig. 5E). These findings suggest that NLS negatively regulates EMT, and significantly attenuates cell migration and invasion *in vitro*.

To further understand the cellular mechanisms underlying our findings, we examined the status of various oncogenic signaling cascades associated with cancer cell survival, proliferation and migration. Among the examined biochemical pathways, we found that inactivation of Akt signaling pathway is notably associated with NLS’s anti-PCa effect. Akt pathway is a typical pathway regulating cell survival, proliferation and migration in human cancer^36^, and is associated with induction of EMT^37^. Akt executes its effects in cells by phosphorylating a variety of downstream substrates, including GSK-3β, Bad, Bim, Bax, Caspase9, p21, p27, and so on^38^. Hyperactivation of Akt pathway has previously been observed in human prostate cancer^39^. GSK3β is one of the major downstream targets of Akt and is involved in migration and proliferation of cancer. The active form of GSK3β is in the dephosphorylated state, when it is phosphorylated by Akt, GSK3β loses its activity^40^. More importantly, GSK-3β negatively regulates the transcription of Snail, a repressor of E-cadherin and an inducer of the EMT. Therefore, inactivation of AKT signaling pathway could promote GSK-3β activity and suppress the expression of Snail, which in turn-impedes the EMT^41^. In our study, we demonstrated that NLS significantly inhibits phosphorylation of Akt, phosphorylation of GSK-3β, and expression of Snail in PCa cells (Figs 3D and 5E). These suggest that NLS may inhibit EMT in PCa cells through attenuating Akt/GSK-3β signaling pathway.

In addition, Akt stabilizes cyclins D1 and E through phosphorylation and inactivation of active GSK-3β, which phosphorylates cyclins D1 and E to stimulate their translocation from nucleus to the cytoplasm for degradation^38^. Akt has also been shown to phosphorylate and directly inhibit p21 and p27, which then inhibit CDK2 and CDK4/6 complexes and promote further G1/S cell cycle transition^42^. The anti-apoptotic function of Akt has been linked to phosphorylation and inactivation of Bad, Bax, Bim and caspase-9, activation of NF-κB, inhibition of cytochrome c release from mitochondria, overexpression of Bcl-2, and so on^43^. In this study, we observed inactivation of Akt and activation of GSK-3β in NLS treated PCa cells (Fig. 3D), which suggest that inhibition of Akt signaling and activation of GSK-3β partially contribute to the pro-apoptotic and G1/S phase arrest activity of NLS in PCa cells.

In summary, our results demonstrated the anti-cancer properties of NLS in PCa cells, both *in vitro* and *in vivo*. NLS inhibits PCa cell growth, migration and invasion through induction of apoptosis, G1/S phase cell cycle arrest and phenotypic inversion of EMT, which is realized by attenuating Akt signaling pathway. Therefore, NLS may serve as a potential adjuvant agent for the treatment of prostate cancer patients.

## Methods and Materials

### Reagents

RPMI 1640, DMEM, fetal calf serum, trypsin, and antibiotics were purchased from Gibco (Grand Island, NY, USA). CellTiter 96AQueous One Solution Cell Proliferation Assay (MTS) were obtained from Promega (Madison, WI, USA). Primary antibodies against Bcl-2, Bax, Bim, Puma, AKT, GSK-3β, PRAP, Caspase-3,7,8,9, P53, P21, P27, and cyclin D1, cyclin E2, CDK2/4/6, β-catenin, E-cadherin, Vimentin, Snail, GAPDH, β-actin etc. were obtained from Cell Signaling Technology (Beverly, MA, USA). Goat anti-rabbit, and anti-mouse and rabbit anti-goat secondary antibodies were the product of Invitrogen (Carlsbad, CA, USA). Polyvinylidene fluoride (PVDF) membrane (Immobilon-P) was purchased from Millipore (Bedford, MA, USA). All other chemicals and reagents were of analytical reagent grade purchased from Sigma (St. Louis, MO, USA).

### Cell lines and culture conditions

Four prostate cancer cell lines PC3, DU145, RM1 and C4-2B and one immortalized human normal prostatic epithelial cell line RWPE-1 were obtained from American Type Culture Collection (ATCC) (Manassas, VA, USA) and were separately cultured in RPMI 1640 (PC3, DU145), DMEM (RM1), T-medium (C4-2B) and K-SFM (RWPE-1) containing 10% fetal calf serum, 100 U/mL penicillin and 100 μg/mL streptomycin at 37 °C and 5% CO_2_.

### Animals and ethics

Nude mice (male, 15.0 ± 2.0 g) of 4–6 weeks old were provided by the Experimental Animal Laboratory of Guangxi Medical University (Nanning, China). The mice were given food and water available ad libitum, and were allowed to acclimatize for one week before the experiments. Experimental conditions and procedures involving animals were approved by Institutional Animal Ethics Committee (IAEC), Guangxi Medical University, and carried out in accordance with laboratory animal use guidelines of IAEC. Animal handling followed the National Animal Welfare Law of China.

### Litchi seed extracts

Litchi seeds were purchased from LBX pharmacy (Nanning, China) and processed at Guangxi Botanical Garden of Medicinal Plants (Nanning, China) to obtain Litchi seed extract. Briefly, dried Litchi seeds were ground to fine powder and then extracted with 75% ethanol at 1:10 (m/v) of solid-liquid ratio, the temperature was 60 °C and lasted for 1.5 h, repeated the procedure 3 times. The obtained crude ethanol extract was filtered through a vacuum Buchner funnel and Whatman No. 1 filter paper. The solvent was evaporated in vacuum to obtain the extract. Suspended the extract at 1:1(m/v) of raw material and deionized water and then extracted successively using petroleum ether (PE), ethyl acetate (EtOAc) and n-butyl alcohol (n-BuOH) on the basis of systematic solvent extraction method^44^. The supernatant and sediment were separated through decompress filtration, the residue was re-extracted. The polarity of extracts obtained was variable from small to large (PE, EtOAc, n-BuOH and Ethanol). Combined the obtained extracted solution and concentrated using a rotary evaporator under reduced pressure, then were freeze-dried. The yield rate of each fraction from crude starting materials was 1.33%, 1.06%, 2.35% and 3.51% (w/w) respectively. The extracts were stored in freezer under −20 °C before used.

### Composition analysis

NLS was analyzed by LC-MS to generate a fingerprint of phytochemicals present in the plant. Powder of NLS (50 mg) was dissolved in 1 ml DMSO, then diluted 50 mg/ml stock to 500 μg/ml with methanol and 5 μl filtered sample was subjected to reverse phase (RP) chromatography both in positive and negative ionization modes. For positive and negative RP separation, samples were chromatographically resolved with WatersXBridgeC18, 3.5 μm, 4.6 × 150 mm column on an LC-20A UFLC system (Shimadzu, Kyoto Japan) with an auto-sampler held at 10 °C, using linear gradient of 0.1% Formic acid in water (A) and 0.1% formic acid in acetonitrile (B) over 40 min. Gradient elution was linearly programmed as follows: 0–1 min 15% (v/v) B, 1–12 min 15B–30%B, 12–18 min 30B–70%B, 18–25 min held at 70B, 25–28 min 70B–95%B, 28–33 min held at 95%B, 33–34 min 95B–15%B and then held to 40 min, at a constant flow rate of 1.0 ml/min. The injection volume was 5 μl. Mass spectra were analyzed on an Applied Biosystem Sciex API 4500 Q-Trap mass spectrometer (Foster City, CA, USA), equipped with a Turbo V™ Ion Source. The ion source was operated in both positive and negative-ion ESI mode with the following flow injection parameters: ion spray voltage: +/−4500 V; source temperature: 550 °C; curtain gas: 40 psi N2; GS1: 50 psi N2; GS2: 50 psi N2; heated interface. MS1 spectra were obtained using the enhanced mass spectrum (EMS) scan with a declustering potential (DP) of ±100 V and an entrance potential (EP) of ±10 V. All MS2 spectra were collected by enhanced product ion (EPI) scan with Q1 set at unit resolution and the triple–quadrupole (QqQ) scanning ranges were set based the target parent ions, with a step size of 0.12 Da (at a rate of 120 Da/s). Data acquisition was carried out with Analyst Software 1.6.2 software.

### Cell proliferation assay

Cell proliferation/viability was determined by MTS assay. Briefly, PC3, DU145, RM1, C4-2B and RWPE-1 cells were seeded at 3,000 cells/well in 96-well plates and allowed to adhere overnight. Then cells were treated with various concentrations (0, 31.25, 46.875, 62.5, 93.75, 125, 187.5, 250 μg/mL) of four fractions for 24, 48 and 72 hours, and DMSO was used as negative control. 20 μL MTS solution was added to each well at the end points and incubated at 37 °C for 2 hours. Absorbance at 490 nm was detected using microplate reader. Cell viability rate (%) = treated group/control group × 100%. The half-maximal inhibitory concentration (IC_50_) was calculated using the Bliss method. Data are the averages of three independent experiments.

### Colony-forming assay

PC3 and DU145 cells were plated at a density of 400 cells per well in 6-well tissue culture plates. 24 hours later, the cultures were treated with NLS (15, 30 and 60 μg/ml) or DMSO for 8 days and medium was replaced with fresh one containing NLS every 4 days. For determination of colony formation, cultures were fixed with 4.0% paraformaldehyde and stained with crystal violet. The number of colonies was counted after scanned with Canon CanoScan 9000F Color Image Scanner. The colony formation rate (%) = number of colonies/number of plated cells × 100%.

### Migration assay

The inhibition of tumor cell migration by NLS was performed by wound-healing assay^45^. Briefly, PC3 and DU145 cells were allowed to grow into full confluence in 6-well plates, and then a vertical wound was created with a 200 μl pipette tip. The cell debris was removed and fresh complete medium with various concentrations of NLS (60 and 120 μg/ml) was added. Cells were incubated at 37 °C and 5% CO_2_ for 24 hours and photographed using a microscope at three time points (0, 12 and 24 hours). The area of migration was measured and analyzed with Image J software and the experiments were conducted thrice.

### Invasion assay

Invasion assay was determined using transwell chambers (Corning New York, NY, USA) with 8 μm pore membranes as described previously^46^. Briefly, after 24, 48 and 72 hours pre-treatment with NLS (30, 60 and 120 μg/ml) in 6-well plate, tumor cells were collected and re-suspended in serum free cell culture media. A total of 5 × 10^4^ cells in 100 μl were added in the upper chamber, and 600 μl of complete medium was added into the bottom of the lower chamber. The cells were allowed to invade for 22 hours at 37 °C and 5% CO_2_. Invaded cells were stained with 0.2% crystal violet and counted in five random microscopic fields. The percentage of inhibition was expressed using control well as 100%. Each assay was performed in triplicate.

### Cell cycle and apoptosis analysis

Cell cycle and apoptosis were analyzed by flow cytometry (FACScan; BD Biosciences) as previously described^47^ with some modifications. In brief, PC3 and DU145 cells were seeded at 2 × 10^5^ cells in 12.5 cm^2^ culture flasks and allowed to grow for 24 h before NLS exposure (60 and 120 μg/ml) or DMSO exposure. Cells were harvested after 24, 48, and 72 h and washed twice with phosphate-buffered saline (PBS). For cell cycle analysis, the treated cells were incubated with propidium iodide (PI) according to the protocol of CycleTEST PLUS DNA Reagent Kit (BD PharMingen, San Diego, CA). Then the stained cells were analyzed by flow cytometry using FL-2A to score the DNA content of the cells. Approximately 30,000 cells were evaluated per sample. The population of each phase was calculated using Modfit software (Verity Software House, Topsham, ME). For the apoptosis analysis, Annexin V-FITC apoptosis detection kit I (BD PharMingen, San Diego, CA) was used to analyze cell apoptosis, following the manufacturer’s protocols. Briefly, the treated cells were stained with Annexin V-FITC and PI for 15 min at room temperature in the dark before flow cytometry analysis. Approximately 50,000 cells were evaluated for each sample. The experiments were conducted thrice, and the results are reported as the mean of the three experiments.

### Western blotting analysis

PC3 and DU145 cells were treated with NLS (120 μg/ml) for 24, 48 and 72 hours, then cells were washed twice with ice-cold DPBS and incubated in lysis buffer in the presence of phenylmethylsulfonyl fluoride, protease inhibitor cocktail, phosphatase inhibitors cocktail 2 and 3 to obtain cell extracts. The protein concentration of the cell extracts was detected by BCA Protein Assay Reagent Kit (Rockford, IL, USA), and 50 μg of protein was loaded to SDS-PAGE gels and were transferred to polyvinylidene fluoride membranes (PVDF). The blots were incubated with the appropriate concentration of specific primary antibody overnight at 4 °C. After washing, the blots were incubated with secondary antibody conjugated with peroxidase and an enhanced chemiluminescence detection system (Rockford, IL, USA) was used for detection. GAPDH or β-actin was used as an equal loading control.

### Tumor xenografts in nude mice and drug treatments

In mouse xenografts, we utilized the luciferase-expressing prostate cancer cell line (PC3^-luc^) for *in vivo* bioluminescence imaging using the IVIS imaging systems (Caliper Life Sciences, Hopkinton, MA, USA). Two million PC3^-luc^ cells were injected subcutaneously into the right flank of each mouse (day 0). After the primary tumors had reached a mean volume of about 50 mm^3^, mice carrying tumor xenografts were arbitrarily divided into 4 groups (n = 10 per group) and treatment was started. Mice were orally treated with NLS (52 and 26 mg/kg) once daily for 24 consecutive days. Paclitaxel (20 mg/kg), used as reference drug for positive control, was administered intraperitoneally once per week. Body weight and tumor size of the mice were monitored every 3 days, and *in vivo* imaging was performed once a week. Tumor volumes (V) were calculated using the formula V = ½ (length × width^2^). During the experiment, the animals did not show any discomfort.

### Statistical analysis

Data were presented as mean ± SDs for at least three repeated individual experiments for each group, and analyzed by one-way ANOVA followed by LSD and Dunnett’s T3 using SPSS version 17.0 software. A value of p < 0.05 was considered statistically significant.

## Additional Information

**How to cite this article**: Guo, H. *et al*. Litchi seed extracts diminish prostate cancer progression via induction of apoptosis and attenuation of EMT through Akt/GSK-3β signaling. *Sci. Rep.* **7**, 41656; doi: 10.1038/srep41656 (2017).

**Publisher's note:** Springer Nature remains neutral with regard to jurisdictional claims in published maps and institutional affiliations.

## Supplementary Material

Supplementary Information

## Figures and Tables

**Table 1:** Relevant analytical data for compounds isolated from NLS.

Peak	RT (min)	Parention	MS/MS	Mol. formula	Proposed compound
1	3.27	163.1 [M-H]^−^	145.9, 118.9, 117.1	C_9_H_8_O_3_	Coumaric acid^48^
2	4.45	139.1 [M + H]^ + ^	121.0, 111.1, 85.0, 69.1	C_7_H_6_O_3_	Protocatechuic aldehyde^49^
3	4.47	289.1 [M-H]^−^	245.2, 220.0, 191.0, 173.0, 162.8, 157.9,	C_15_H_14_O_6_	(-)-Epicatechin^48^
4	5.67	417.1 [M-H]^−^	371.0, 255.1, 160.9	C_21_H_38_O_8_	Pterodontriol-D-6-O-β-D-glucopyranoside^17^
5	6.30	609.2 [M-H]^−^	301.1, 271.1, 255.0, 151.1	C_27_H_30_O_16_	rutin^50^
	6.72	611.2 [M + H]^+^	465.2, 356.0, 345.0, 303.0		
6a*	6.30	575.2 [M-H]^−^	539.0, 449.2, 423.1, 407.2, 289.1, 285.1	C_30_H_24_O_12_	2α,3α-Epoxy-5,7,3′,4′-tetrahydroxyflavan-(4β-8-catechin)^17^; 2α,3α-Epoxy-5,7,3′,4′-tetrahydroxyflavan-(4β-8-epicatechin)^17^; 2β,3β-Epoxy-5,7,3′,4′-tetrahydroxyflavan-(4α-8-epicatechin)^17^
6b*	7.27				
6c*	8.42				
6a*	6.62	577.2 [M+H]^+^	559.2, 437.2, 425.2, 419.0, 299.0, 287.0		
6b*	7.85				
6c*	9.28				
7	7.79	623.2 [M-H]^−^	577.4, 533.3, 461.3, 427.2, 315.1, 300.1	C_10_H_8_O_4_	Tamarixetin3-O-rutinoside^11^
8	9.33	405.2 [M-H]^−^	301.2, 239.2, 225.4, 220.0	C_19_H_34_O_9_	Litchioside C^18^
9	9.97	435.1 [M-H]^−^	417.2, 341.0, 315.1, 273.1,		
167.0, 123.2	C_21_H_24_O_10_	Phlorizin^51^			
10a*	12.01	563.3 [M-H]^−^	443.3, 357.1, 339.1, 297.1, 269.2, 255.1	C_27_H_32_O_13_	(2S)-Pinocembrin-7-O-(6-O-α-L-rhamnopyranosyl-β-D-glucopyrano-side)^19^; (2R)-Pinocembrin-7-neohesperidoside^17^
10b*	12.88				

^*^Isomers which can’t be identified solely by LC-MS/MS.
