# Author Correction: Litchi seed extracts diminish prostate cancer progression via induction of apoptosis and attenuation of EMT through Akt/GSK-3β signaling

**DOI:** 10.1038/s41598-021-94623-5

**Published:** 2021-07-20

**Authors:** Hongwei Guo, Hua Luo, Hebao Yuan, Yudui Xia, Pan Shu, Xin Huang, Yi Lu, Xia Liu, Evan T. Keller, Duxin Sun, Jiagang Deng, Jian Zhang

**Affiliations:** 1 Center for Translational Medicine, Guangxi Medical University, 22 Shuangyong Road, Nanning, 530021 China; 2 Key Laboratory of Longevity and Aging-Related Disease, Chinese Ministry of Education, 22 Shuangyong Road, Nanning, 530021 China; 3 College of Pharmacy, Guangxi University of Chinese Medicine, 179 Mingxiu Dong Road, Nanning, 530001 China; 4 Department of Pharmaceutical Sciences, College of Pharmacy, University of Michigan, 1600 Huron Parkway, Ann Arbor, MI 48109 USA; 5 Xinxiang Central Hospital of Henan, 56 Jinsui Road, Xinxiang, 453000 China; 6 Department of Urology and Pathology, School of Medicine, University of Michigan, 2800 Plymouth Road, Ann Arbor, MI 48109 USA; 7 Southern University of Science and Technology, School of Medicine, 1088 Xueyuan Blvd., Nanshan District, Shenzhen, 518055 China

Correction to: *Scientific Reports* 10.1038/srep41656, published online 30 January 2017

This Article contains errors. As a result of a mistake during the figure assembly, in Figure 5A panel DU145/0 h/120 μg/ml NLS was a duplication of a panel DU145/0 h/60 μg/ml NLS, and panel PC3/24 h/60 μg/ml NLS was a duplication of a panel DU145/24 h/60 μg/ml NLS. The corrected Figure 5 appears below as Figure 1.

**Figure 1:**
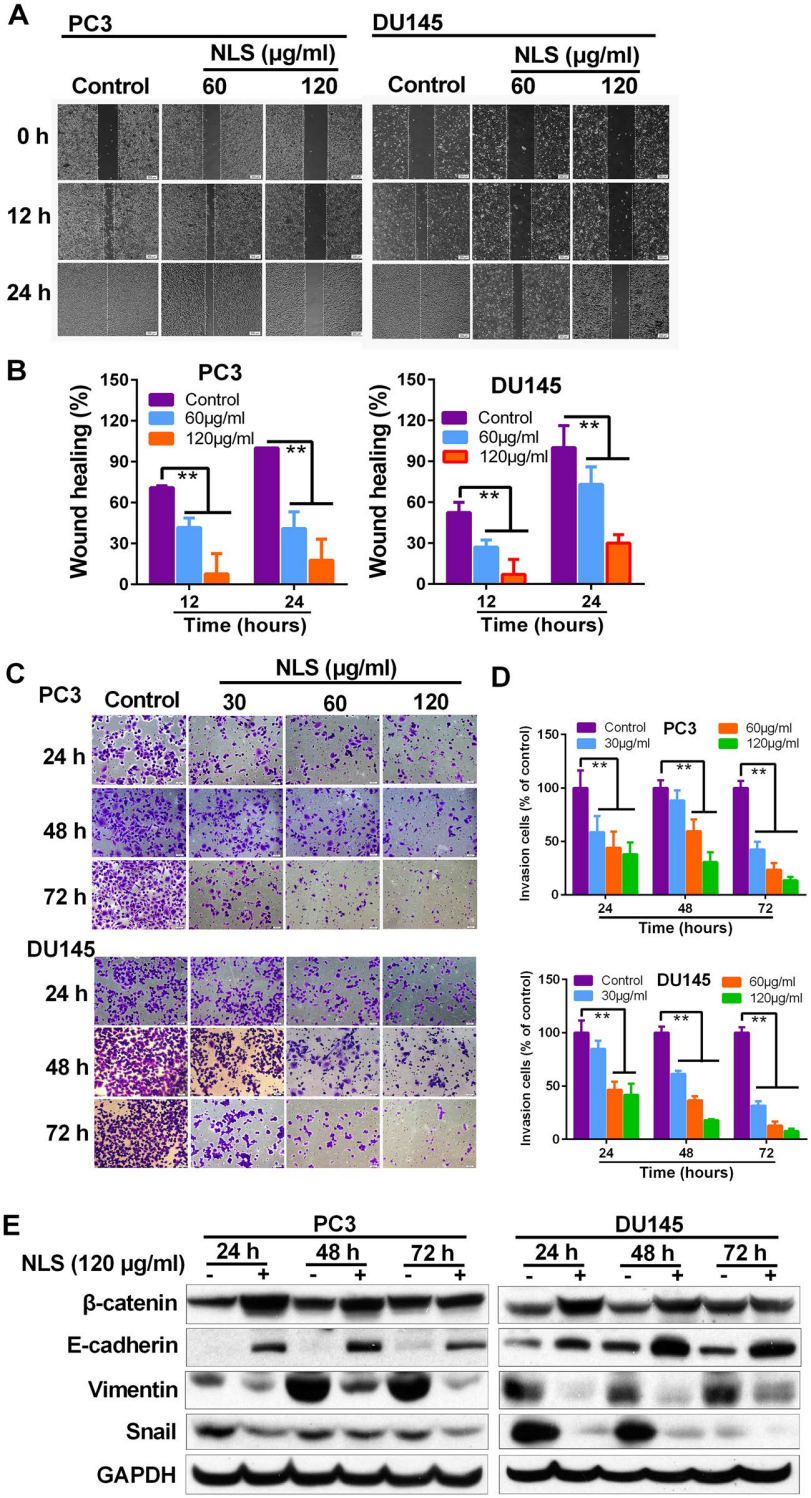
(**A**) Wound-healing assay was used to examine cellular migration. PC3 and DU145 cells were allowed to grow into full confluence in 6-well plates, and then a wound was created with a pipette tip. NLS was added to the well and images were obtained using a microscope at 0, 12 and 24 h. Three independent experiments were examined and representative images were presented. (**B**) Quantification of the average wound healing degree of PC3 and DU145 cells. (**C**) Invasiveness of PC3 and DU145 cells that underwent NLS treatment was determined in transwell invasion assay. PC3 and DU145 cells, after 24, 48 and 72 h pretreatment with NLS (30, 60 and 120 μg/ml), were added in the top chamber and allowed to invade for 22 h. Crystal violet-stained cells represent the fraction of cells that migrated from the top to the bottom chamber of the membrane. Three independent experiments were examined and representative images were presented. (**D**) Quantification of invaded PC3 and DU145 cells in the bottom chamber. Data are expressed as mean ± SD. Compared with control group: *p < 0.05, **p < 0.01. (**E**) Expression of EMT-related proteins in PC3 and DU145 cells was determined by western blotting.

These changes do not affect the conclusions of the article.

