# Supplementary material for: Litchi seed extracts diminish prostate cancer progression via induction of apoptosis and attenuation of EMT through Akt/GSK-3β signaling
Source: Sci Rep. 2017 Jan 30;7:41656. doi: 10.1038/srep41656 (PMC5278538; doi:10.1038/srep41656)
Supplement: Supplementary Information [file srep41656-s1.doc]

**Litchi seed extracts diminish prostate cancer progression via induction of apoptosis and attenuation of EMT through Akt/GSK-3β signaling**

Hongwei Guo^1,2*^, Hua Luo^3,*^, Hebao Yuan^5*^, Yudui Xia^1,2,4^, Pan Shu^5^, Xin Huang^1,2^, Yi Lu^1,2^, Xia Liu^1,2^, Evan T. Keller^6^, Duxin Sun^5^, Jiagang Deng^3^&Jian Zhang^1,2,6,7^

^1^ Center for Translational Medicine, Guangxi Medical University, 22 Shuangyong Road, Nanning 530021, China.

^2^ Key Laboratory of Longevity and Aging-related Disease, Chinese Ministry of Education, 22 Shuangyong Road, Nanning 530021, China.

^3^College of Pharmacy, Guangxi University of Chinese Medicine, 179 Mingxiu Dong Road, Nanning 530001, China.

^4^Xinxiang Central Hospital of Henan, 56 Jinsui Road, Xinxiang 453000, China.

^5^Department of Pharmaceutical Sciences, College of Pharmacy, University of Michigan, 1600 Huron Parkway, Ann Arbor, MI 48109, USA.

^6^ Department of Urology and Pathology, School of Medicine, University of Michigan, 2800 Plymouth Road, Ann Arbor, MI 48109, USA

^7^ Southern University of Science and Technology, School of Medicine, 1088 Xueyuan Blvd., Nanshan District, Shenzhen, 518055, China

*

These authors contributed equally to this work. Correspondence and requests for materials should be addressed to

Y.S. (email: yangsun111@126.com) or W.Y. (email: yiweifmmu@126.com)

*

These authors contributed equally to this work. Correspondence and requests for materials should be addressed to

Y.S. (email: yangsun111@126.com) or W.Y. (email: yiweifmmu@126.com)

These authors contributed equally to this work. Correspondence and requests for materials should be addressed to

Y.S. (email: yangsun111@126.com) or W.Y. (email: yiweifmmu@126.com)

These authors contributed equally to this work. Correspondence and requests for materials should be addressed to

Y.S. (email: yangsun111@126.com) or W.Y. (email: yiweifmmu@126.com)

These authors contributed equally to this work. Correspondence and requests for materials should be addressed to

Y.S. (email: yangsun111@126.com) or W.Y. (email: yiweifmmu@126.com)

These authors contributed equally to this work. Correspondence and requests for materials should be addressed to

Y.S. (email: yangsun111@126.com) or W.Y. (email: yiweifmmu@126.com)

These authors contributed equally to this work. Correspondence and requests for materials should be addressed to

Y.S. (email: yangsun111@126.com) or W.Y. (email: yiweifmmu@126.com)

These authors contributed equally to this work. Correspondence and requests for materials should be addressed to

Y.S. (email: yangsun111@126.com) or W.Y. (email: yiweifmmu@126.com)

* These authors contributed equally to this work.

Correspondence and requests for materials should be addressed to D.S. ([duxins@med.umich.edu](mailto:duxins@med.umich.edu)),

J.D. ([dengjg53@hotmail.com](mailto:dengjg53@hotmail.com) ) or J.Z.([jianzhang008@hotmail.com](mailto:jianzhang008@hotmail.com) )

**
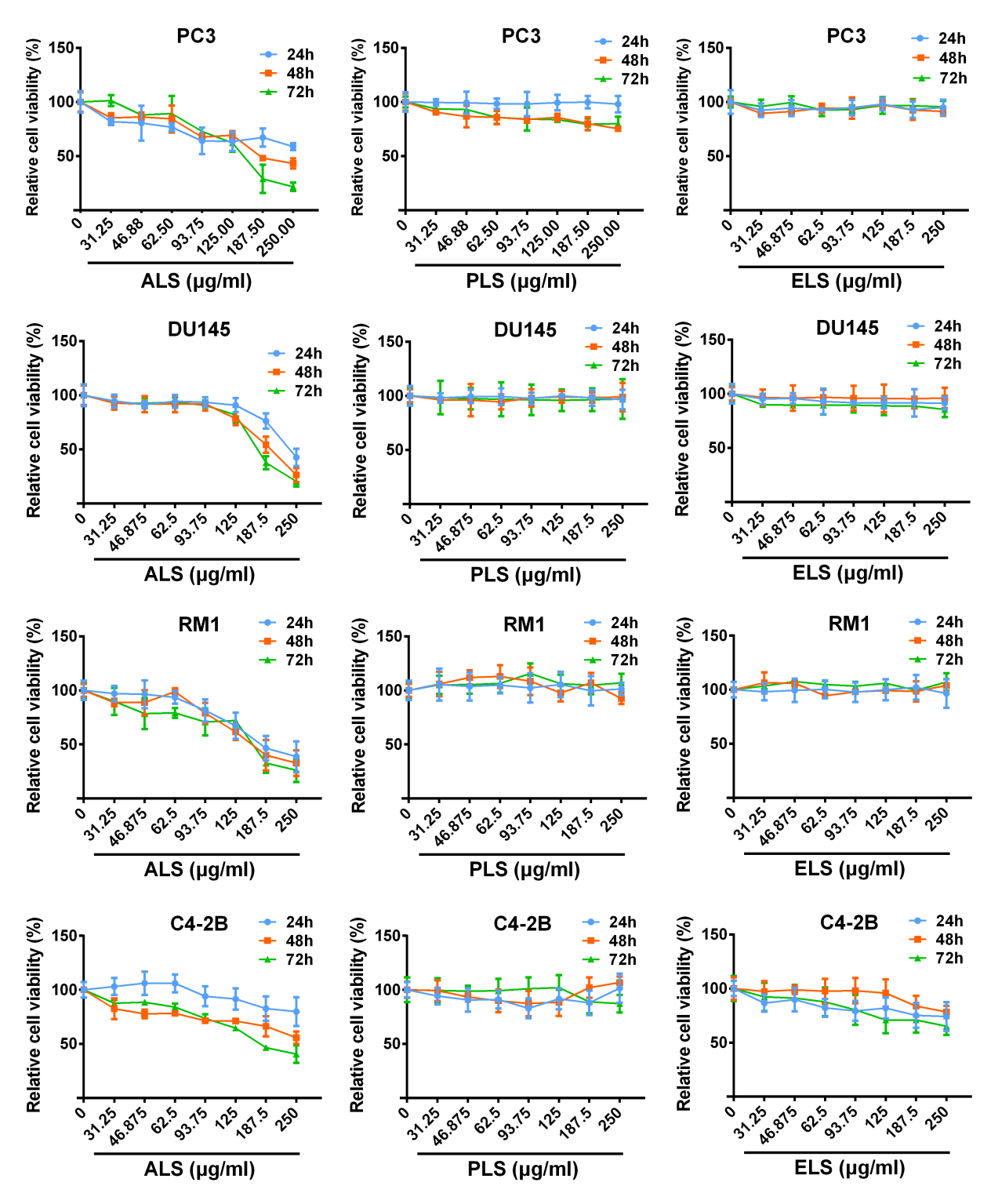
**

**Supplementary Figure S1. Effect of various fractions of Litchi seed extract on the viability of prostate cancer cells.** Prostate cancer cell lines PC3, DU145, C4-2B and RM1 were treated with various fractions of Litchi seed extract at concentrations of 0 to 250 μg/ml in triplicates for 24, 48 and 72 h. Cell viability was measured by MTS assay. ALS: ethyl acetate extract of Litchi seed; PLS: petroleum ether extract of Litchi seed; ELS: 75% ethanol extract of Litchi seed.
